# The Pay Hospital System in Great Britain

**Published:** 1917-04-28

**Authors:** 


### THE PAY HOSPITAL SYSTEM IN GREAT BRITAIN

### It Should Become Widespread After the War.*

The war has revolutionised many things and materially altered the outlook on life of probably every class of worker throughout the nation and Empire. The Empire has already largely adopted the system of pay wards and pay hospitals. The Old Country has halted and shuffled, and kept the modern hospital handicapped unnecessarily and unwisely through the force of tradition and the failure to appreciate the wholesome wish of an ever-increasing number of persons to obtain on payment, in whole or in part, hospital treatment for themselves and their families. After the war a revolution in hospital provision may be confidently expected, and it will certainly include the prompt establishment throughout the country of various types of pay wards and hospitals. It should be possible to grade it, that it may be brought within the means and requirements of every class of the community. There will still be free beds in adequate numbers to provide those members of the population whose circumstances entitle them to receive free treatment; but the recognition by the public of the manifest fact, that to-day every person requiring surgical and many other forms of treatment, will best find it in the supremest excellence within the walls of a modern, up-to-date hospital, will be given substance to by the provision of such accommodation for all classes. How exactly the provision will be made we have not space to demonstrate in this article. Let it suffice to say that after the war an opportunity will arise to provide everywhere for the accommodation of paying patients under the best possible conditions at the minimum expenditure without delay.

The first pay hospital in this country, which has been established and working for nearly forty years with marked success, with the approval, support and generous recognition of the leading members of the medical profession, illustrates the simplicity of hospital finance on a business basis. Originally founded by the generous contributions of a relatively few earnest persons of many professions, and representative leaders of public opinion, the Home Hospital, Fitzroy House, Fitzroy Square, after twenty-two years' experience, had to face the problem of rebuilding and enlargement. Its policy had always been to leave the patients to make their own arrangements as to fees with the members of the medical profession, and the charge for residence and maintenance was always kept to a sum which did not exceed by more than 10 per cent. the actual cost entailed. The object of the founders was not to make money, but to show that money could be made, if necessary, to maintain a pay hospital in the highest efficiency and, should occasion arise, to extend and improve it. When the hour for rebuilding and extension arrived it was found possible to make a business arrangement for the advance of the necessary funds to meet the capital

* First article appeared on April 21, 1917, page 47.

sum entailed to complete the work, and to provide for its repayment by annual instalments extending over a period of years. This plan has been pursued for fourteen years with the most satisfactory results; the capital has already been nearly repaid, and the Home Hospital has been maintained in full working and excellence, whilst its position was never better or its reputation higher, or its popularity and the demand upon its beds greater than it is at the present moment.

Its popularity and usefulness may be gathered from the circumstance that eighty-five practitioners in London had patients in this hospital during the year 1916. They were practically all surgical cases requiring operation. The average number of patients throughout the year was twenty-five; the average stay of each patient being 17½ days; 523 patients were admitted, and the average cost per patient was under £1 per diem. Thus the founders of the first pay hospital in the United Kingdom, after forty years' devoted work and the enforcement of business management of the highest excellence, have justified the confidence of those who fought a most strenuous and difficult battle at the outset. They have proved to demonstration the soundness of the views they submitted to the public at the meeting at the Mansion House, when the pay hospital was approved by the public and the profession. The way is thus paved to make the general introduction of the pay hospital system throughout the United Kingdom after the war a simple business proposition, which will do much to render the life of sick and injured people of all classes in this country, immeasurably, happier and better than it was possible to make it for the majority of them under the old system.

